# Whipped Cream Regulation by Hydrophilic Sucrose Esters: Interfacial Behavior and Whipping Properties

**DOI:** 10.3390/foods15172995

**Published:** 2026-08-26

**Authors:** Di Zeng, Cuiling Li, Lihua Huang, Junwei Wang, Yongjian Cai, Qiangzhong Zhao, Mouming Zhao

**Affiliations:** 1 School of Food Science and Engineering, Guangdong Ocean University, Yangjiang Campus, Yangjiang 529500, China; dickzengen@163.com (D.Z.); licuiling04@163.com (C.L.); jweiwangg@163.com (J.W.); 2 School of Food Science and Engineering, South China University of Technology, Guangzhou 510641, China; hlh5213@163.com (L.H.); femmzhao@scut.edu.cn (M.Z.); 3 School of Food Science and Bioengineering, Changsha University of Science and Technology, Changsha 410114, China

**Keywords:** sucrose esters, interfacial rheology, whipped cream, partial coalescence

## Abstract

Hydrophilic sucrose esters are widely used in whipped cream, but the relationship between their molecular structure, interfacial behavior, and whipping performance remains unclear. This study investigated three hydrophilic sucrose esters, S1170, S1570, and P1570, in sodium caseinate-stabilized cream systems. Interfacial measurements showed that sucrose ester concentration strongly affected the adsorption behavior and viscoelasticity of the oil/water interface. Low concentrations produced rheological responses consistent with the formation of a more elastic mixed interface, whereas excessive addition was associated with competitive adsorption, reduced interfacial protein coverage, and weakened interfacial viscoelasticity. The molecular structure of sucrose esters further influenced this process: S1170, with a higher polyester content, showed behavior consistent with stronger interfacial interactions, while P1570 showed a greater decrease in interfacial modulus. These interfacial differences were associated with changes in whipping behavior. At 0.10 wt%, sucrose esters slowed fat partial coalescence, prolonged the optimal whipping time, and increased overrun. At 0.50 wt%, they accelerated early-stage coalescence and increased serum loss, especially for P1570. These findings suggest that selecting appropriate sucrose ester type and dosage is essential for balancing interfacial stability, fat partial coalescence, and whipped cream quality.

## 1. Introduction

Whipped cream is a typical aerated emulsion system whose texture, overrun, and stability are governed by the dynamic formation of fat–air networks during whipping [1,2]. Among the factors affecting whipping performance, fat partial coalescence plays a central role because it determines the formation of the semi-solid network required to stabilize incorporated air bubbles [3,4]. However, the whipping process involves complex coupling between air incorporation, interfacial destabilization, and fat crystal interactions, making it difficult to achieve controllable and reproducible whipping behavior, particularly when formulation variables are adjusted for product design [5].

Low-molecular-weight emulsifiers (LMWEs) are widely used in whipped cream systems because they can regulate fat partial coalescence by modifying the interfacial properties of fat globules [6]. These emulsifiers adsorb at the oil/water interface and interact with proteins, thereby influencing interfacial tension, viscoelasticity, and interfacial film stability during whipping [7]. Previous studies have demonstrated that emulsifier–protein interactions affect fat crystallization, interfacial film strength, and foam stability [8]. In particular, the competitive and cooperative adsorption between proteins and small molecule emulsifiers can generate different interfacial structures, ranging from protein-dominated films to mixed adsorption layers and emulsifier-dominated interfaces [9,10]. These transitions are expected to strongly influence interfacial viscoelasticity and molecular exchange dynamics, which are closely associated with fat partial coalescence behavior during whipping [11,12]. Interfacial dilatational rheology provides an effective approach for characterizing these interfacial behaviors and establishing relationships between interfacial properties and whipping performance [13,14]. Nevertheless, the mechanisms linking interfacial adsorption behavior and macroscopic whipping properties remain insufficiently understood.

Sucrose esters are important nonionic emulsifiers widely used in whipped cream formulations. Their physicochemical properties can vary considerably depending on fatty acid composition and esterification degree, resulting in different interfacial behaviors and whipping performances [4,15]. Previous studies have shown that sucrose esters can influence fat crystallization, interfacial structure, and foam stability in whipped cream systems [7,16,17]. Our previous work demonstrated that hydrophilic sucrose ester S1570 could regulate whipping behavior through competitive adsorption with proteins at the interface [18]. However, previous studies have mainly focused on individual sucrose esters, HLB values, fat crystallization, or final whipping performance, while the separate effects of esterification degree and fatty acid composition on protein–emulsifier interfacial behavior remain unclear. By comparing S1170 with S1570 and S1570 with P1570, the present study links sucrose ester structure with interfacial rheology, fat partial coalescence, and whipped cream quality.

Therefore, this study investigated the effects of different hydrophilic sucrose esters with varying esterification degrees and fatty acid compositions on the interfacial properties and whipping behavior of cream systems. Interfacial dilatational rheology was used to characterize the interactions between sucrose esters and sodium caseinate at the oil/water interface. Meanwhile, emulsion properties, fat partial coalescence behavior, and whipped cream quality were evaluated to establish the relationship between interfacial viscoelasticity and macroscopic whipping performance. This work aims to provide an interfacial engineering perspective for understanding and regulating whipped cream systems.

## 2. Materials and Methods

### 2.1. Materials

Sucrose esters S1170 (HLB 11, Sucrose monoester 55%, stearic acid 70%), S1570 (HLB 15, Sucrose monoester 70%, stearic acid 70%) and P1570 (HLB 15, Sucrose monoester 70%, palmitic acid 70%) were procured from Mitsubishi Chemical Co. (Tokyo, Japan). Sodium caseinate (≥95 wt.% purity) was sourced from Fonterra Cooperative Group (Auckland, New Zealand). Compound stabilizers, including Hydroxypropyl methylcellulose (methyl substitution: 1.9; hydroxypropyl substitution: 0.23; molecular weight: 90,000 Da), xanthan gum (6,000,000 Da), carrageenan (Kappa), and guar gum, were supplied by Guangdong Wenbang Biotechnology Co. (Zhaoqing, China). Corn syrup (solid content > 75%; DE: 40%) was obtained from Zhaoqing Huanfa Biotechnology Co. (Zhaoqing, China), while sucrose came from Dongguan Donta Group Co., Ltd. (Dongguan, China) and glucose from Zhucheng DongXiao Biotechnology Co. (Zhucheng, China). Hydrogenated palm kernel oils (HYSOC-41 HPKO: 51.1 g/100 g C12:0, 15.3 g/100 g C14:0, 8.9 g/100 g C16:0, 17.3 g/100 g C18:0) were generously donated by Ligao Foods Ltd. (Guangzhou, China), and corn oil was purchased from COFCO Fulinmen Food Marketing Co., Ltd. (Tianjin, China). Sigma Florisil (60–100 mesh, Sigma-Aldrich, St. Louis, MO, USA) was acquired from Merck China Ltd. (Nantong, China).

### 2.2. Interfacial Properties

The oil/water interfacial tension was measured using a drop shape analyzer (DSA100, KRÜSS GmbH, Hamburg, Germany) equipped with oscillating drop system. To maintain the oil phase in a liquid state during the measurements, corn oil was used in place of HPKO, as suggested by MacWilliams et al. [19]. Prior to the experiment, polar impurities in the corn oil were removed using Sigma Florisil, ensuring that the oil/water interfacial tension remained within the range of 28.5 ± 0.5 mN/m.

#### 2.2.1. Interfacial Tension

The interfacial tension (*γ*) was calculated by applying the Young–Laplace equation (Equation (1)) to the drop profile:

(1) $$\gamma \;=\;\Delta p(\frac{1}{r_{1}}\;+\;\frac{1}{r_{2}})$$

where *r*_1_ and *r*_2_ are the principal radii of curvature, and *Δp* represents the density difference between water and corn oil.

The sucrose esters (S1170, S1570, and P1570) and protein solutions were prepared separately and then mixed to obtain the desired concentration (0.50 wt% sodium caseinate and varying concentrations of sucrose esters). A drop of the protein–surfactant mixture (approximately 7 μL) was carefully dispensed into an optical glass cuvette containing purified oil and allowed to remain at the needle tip for 7200 s to enable sample adsorption at 25 °C. The interfacial tension and dilatational values were automatically calculated by the inbuilt Kruss Advance software (v1.14).

#### 2.2.2. Interfacial Rheology

Interfacial dilatational rheology was employed to measure the interfacial viscoelasticity of the sucrose ester–sodium ester-sodium caseinate interface at 25 °C. A sinusoidal periodic dilation was applied to the droplet, and the interfacial dilational modulus *E* was calculated based on the periodic changes in the drop’s surface area A and interfacial tension γ during oscillation (Equation (2)):

(2) E = dγ/dlnA

The interfacial dilational modulus E is composed of both the interfacial elastic modulus *E*′ and the interfacial viscous modulus *E*″, which can be expressed as (Equation (3)):

(3) $$E\;=\;E^{\prime }\;+\;iE^{\prime \prime }\;=\;|E|cos\varphi \;+\;i|E|sin\varphi$$

where *φ* represents the phase angle between stress and strain. The elastic modulus *E*′ corresponds to the elastic response of the system, reflecting molecular interactions at the interface. Conversely, the viscous modulus *E*″ indicates the rate at which interfacial tension returns to its original state after deformation, relating to the exchange of emulsifier molecules between the interface and the liquid phase.

In the experiment, an oscillation frequency (0.02–0.20 Hz) and amplitude (5–20%) were chosen to measure sucrose esters–sodium caseinate interfacial rheology at 25 °C.

#### 2.2.3. Lissajous Plots

Lissajous plots can be used to further investigate the effect of droplet deformation on the interfacial layer [20,21]. By plotting the interfacial pressure *π* (*π* = *γ* − *γ*_0_) against the relative interfacial area deformation *ΔA* (*ΔA* = *A* − *A*_0_), the influence of deformation on the interfacial layer can be analyzed.

### 2.3. Emulsion Properties

#### 2.3.1. Emulsion Preparation

The sucrose ester concentrations of 0.10 and 0.50 wt% were selected based on our previous studies [22] and formulation experience. The lower concentration represents a moderate addition level at which sodium caseinate remains an important interfacial component, whereas the higher concentration was selected to examine the effects of more pronounced competitive adsorption on interfacial properties and whipping performance.

The detailed formulations of all treatments are summarized in Table 1. Sodium caseinate (0.50 wt%), stabilizers (0.40 wt%), and sucrose esters (0.10 or 0.50 wt%) were blended with HPKO (14.0 wt%) at 65 °C. Corn syrup (6.2 wt%), sucrose (5.0 wt%), and glucose (18.0 wt%) were dissolved in distilled water (65.0 wt%) at 65 °C. The oil and water phases were then pre-emulsified at 750 rpm for 30 min using a digital stirrer (RW 20, IKA-Werke GmbH & Co. KG, Staufen, Germany) at 65 °C. Distilled water was added to complete the macro-emulsion before homogenization. The macro-emulsions were homogenized twice using a high-pressure homogenizer (APK-100, SPX Flow Technology Danmark A/S, Kolding, Denmark), followed by cooling to 15 °C and hardening at −18 °C for a minimum of 12 h. Before further analysis, samples were thawed to 4 °C.

#### 2.3.2. Droplet Size

The particle size distribution of the emulsions was measured using a Malvern Mastersizer 2000 laser particle sizer (Malvern, United Kingdom). The refractive indices for the dispersed phase, absorbance, and continuous phase were set to 1.414, 0.001, and 1.330, respectively. The emulsion was gradually added to a plastic container until the obscuration reached 10–20%. After 30 s of agitation at 2000 rpm, the measurement commenced.

#### 2.3.3. Interfacial Protein Concentration

The interfacial protein concentration in the emulsion was determined following the method outlined in our previous work [18]. Emulsion samples were centrifuged for 30 min at 10,000× *g* and 4 °C. The supernatant was then carefully removed using a syringe, and this process was repeated until no fat remained in the supernatant. The protein content of both the emulsion and supernatant was measured using the Kjeldahl method, with nitrogen content set to 6.38. The interfacial protein concentration (C, mg/m^2^) was calculated using Equation (4):

(4) C = (Me−Mc)/(Fm×SSA)×1000

where Me represents the protein content in the emulsion (g), Mc is the protein content in the continuous phase (g), Fm is the mass of the dispersed phase (g), and SSA (m^2^/g) is the specific surface area provided by the Mastersizer 2000 software through particle size analysis. The complete separation, protein determination, and calculation procedures were independently repeated three times for each treatment. The results are expressed as mean ± standard deviation (*n* = 3), with the standard deviation representing the uncertainty of the determination.

#### 2.3.4. Adsorption of Fat Droplets on the Air/Water Interface

The adsorption and diffusion of fat globules at the air/water interface are crucial factors determining the rate of surface-mediated partial coalescence. Before measurement, the emulsion was centrifuged at 10,000× *g* and 4 °C for 30 min, and the supernatant was carefully removed using a syringe. This process was repeated several times until no fat was detected. To assess the adsorption of fat globules at the air/water interface, the emulsion was injected beneath the supernatant, and the change in interfacial tension was measured before and after injection.

For the measurement, 25 mL of the supernatant was added to a cylindrical glass dish and left to stand for 30 min. The air/water interfacial tension of the supernatant was then measured using a Dataphysics tensiometer DCAT 21 (DataPhysics Instruments GmbH, Filderstadt, Germany) equipped with a PT11 Wilhelmy platinum plate (10.0 × 19.9 × 0.2 mm). Data were collected at a frequency of 1 s^−1^ over a period of 600 s. After 600 s, 5 μL of emulsion was injected beneath interface using the Dataphysics ESr-LDU injection module, and the change in interfacial tension was continuously monitored.

### 2.4. Whipping Properties

#### 2.4.1. Whipping Process

The emulsion was whipped using a KM060 all-purpose chef machine set to 160 rpm (5th gear) until the optimal whipping time (t_op_) was reached. The determination of the t_op_ was based on previous studies [23]. At this point, small waves form on the surface of the whipped cream and remain unbroken. When the whipping process is stopped at the t_op_, the cream adheres to the mixing head.

#### 2.4.2. Partial Coalescence During Whipping

The degree of fat partial coalescence was evaluated by analyzing the particle size distribution of the cream at different whipping times (1, 3, 5 min, and t_op_). The particle size distribution was measured as described in Section 2.3.2. The degree of partial coalescence was quantified by calculating the area of the second large-size peak formed during the whipping process as a percentage of the total area.

#### 2.4.3. Overrun

The overrun of whipped cream was determined by calculating the change in weight of the emulsion before and after whipping, while maintaining the same volume. The overrun percentage was calculated using Equation (5):

(5) Overrun(%)=(M1/M2)×100

where M_1_ represents the weight of the emulsion before whipping, and M_2_ is the weight of the whipped cream at the same volume.

#### 2.4.4. Firmness

The firmness of the whipped cream samples was assessed using a TA-XT2i Texture Analyzer (Stable Microsystems, Surrey, UK) fitted with an AE/B probe (35 mm diameter). The samples were placed in cylindrical containers (50 mm diameter), and the probe was inserted into the whipped cream at a rate of 1 mm/s until reaching a depth of 15 mm. The firmness was quantified by measuring the force (N) required to penetrate the sample to the specified depth.

#### 2.4.5. Microstructure

The microstructure of the whipped cream was observed using a light microscope (ML31, Guangzhou Micro-shot Technology Co., Ltd.,; Guangzhou, China). The whipped cream was first applied to a depression slide and then covered with a coverslip before being examined under the microscope.

#### 2.4.6. Stabilities

The stability of whipped cream was assessed by evaluating serum loss and the appearance of the cut surface.

Whipped cream (50 ± 5 g) was placed into a plastic funnel set atop a conical flask, then stored in an incubator at 25 °C for 24 h. The weight of the whipped cream was measured before and after storage, and the serum loss percentage was calculated using Equation (6):

(6) Serum loss (%)=[(Mi−Ms)/Mi]×100

where Mi represents the initial weight of the whipped cream, and M*_s_* is the weight after storage.

Additionally, small piles of whipped cream were formed and stored at 25 °C for 12 h. After storage, the piles were swiftly cut with a spatula, and the appearance of the cut surfaces was captured using a digital camera.

### 2.5. Statistical Analyses

Statistical analyses were carried out using Microsoft Excel 2022 and Origin 2021, with all tests conducted in triplicate and the results expressed as means ± standard deviation. The significance of the differences among the data was determined by one-way analysis of variance (one-way ANOVA) using SPSS software (version 17.0). The *p*-values obtained were reported, with a *p*-value of less than 0.05 considered statistically significant.

## 3. Results and Discussion

### 3.1. Interfacial Properties

The phenomenon of fat partial coalescence during whipping serves as the foundation for the internal network structure within the whipped cream. This process involves the penetration of internal fat crystals through the interfacial layer of fat globules. Therefore, a thorough investigation into the properties of the oil/water interface holds significant importance for evaluating the quality of whipped cream [13,24].

In the whipped cream systems under examination within this paper, sodium caseinate (SC) and hydrophilic sucrose esters (SE) constitute the primary components of the interfacial layer surrounding the fat globules [25]. Here, the drop shape method was employed to explore the oil/water interfacial tension and interfacial viscoelasticity of the SE-SC system. Despite inherent disparities between the simulated emulsifiers’ oil/water interfacial properties analyzed through the drop shape method and those of the actual emulsion system, the outcomes nonetheless offer valuable insights into the oil/water interfacial properties within the authentic system [6,23]. Thus, these findings of this part serve as a significant reference point for understanding and optimizing the oil/water interfacial characteristics crucial for whipped cream quality.

#### 3.1.1. Interface Tension

**Figure 1:**
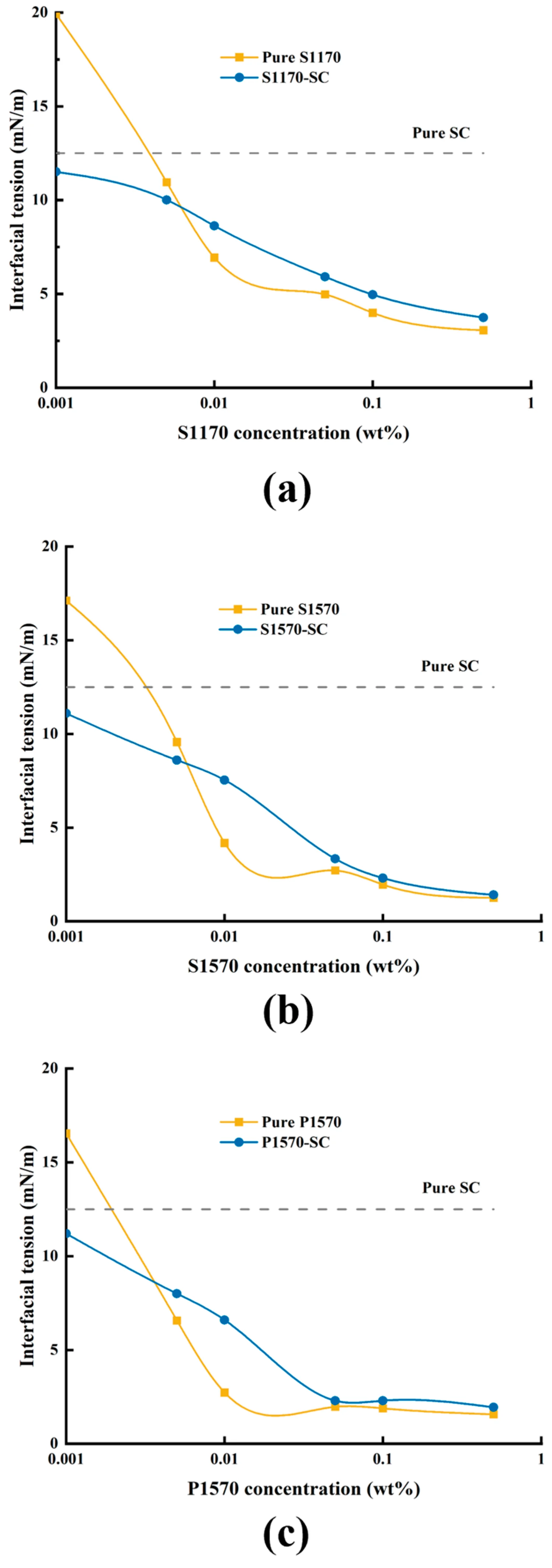
The effect of sucrose esters concentrations and types ((**a**) S1170, (**b**) S1570 and (**c**) P1570) on oil/water interfacial tension.

The data presented in Figure 1 indicate the values of interfacial tension at adsorption equilibrium at the oil/water interface for both the SE alone and SE-SC composite systems. It is observed that in the SE alone system, P1570 demonstrates a stronger capability in reducing γow, with a value of 2.63 mN/m at a concentration of 0.010 wt%, whereas S1170 exhibits a weaker ability, with a value of 6.93 mN/m at the same concentration.

Cabrerizo-Vilchez et al. [26] noted in their study on the interfacial activity of different sucrose esters that monoesters of sucrose palmitate possess high interfacial activity, while polyesters exhibit weak interfacial activity. This discrepancy in interfacial tension reduction among the three sucrose esters could be attributed to their fatty acid composition and esterification degree. Specifically, P1570, containing more short-chain sucrose palmitate monoesters, displays stronger interfacial tension reduction, whereas S1170, containing more polyesters, exhibits weaker interfacial tension reduction. This highlights the significance of the chemical composition and structure of sucrose esters in determining their interfacial activity.

When the concentration of sucrose esters was below 0.005 wt%, the interfacial tension (γow) of the SE-SC composite system was lower compared to both the SE alone and SC alone systems. This reduction occurred because sucrose ester and sodium caseinate co-adsorbed at the oil/water interface, leading to a decrease in γow. As the sucrose ester concentration exceeded 0.010 wt%, the γow of the SE-SC system became intermediate between that of the SE alone and SC alone systems. This indicates competitive adsorption of both components at the interface. Upon further increasing the sucrose ester concentration to approximately 0.10 wt%, the γow of the SE-SC system approached that of the SE alone system. This suggests that sucrose ester predominates at the interface at this concentration, resulting in the SE-SC system exhibiting properties similar to those of the SE alone system.

#### 3.1.2. Effect of Sucrose Ester Concentration on the Interfacial Rheological Properties

In this part, the interfacial dilatational rheological properties of the S1570-SC system were investigated at various concentrations of S1570. This involved conducting frequency sweeping, amplitude sweeping, and Lissajous-plot curves.

##### Frequency Sweeping

**Figure 2:**
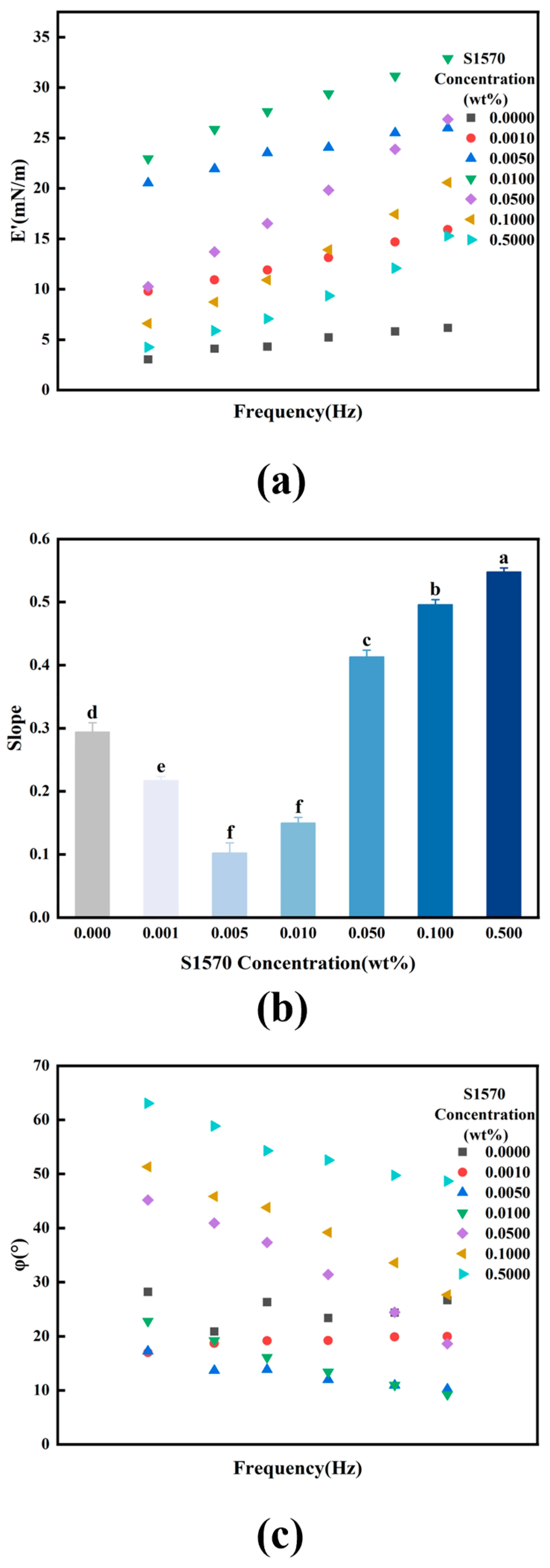
Variation of dilatational complex modulus (**a**) and loss angle (**c**) with frequency for the stable oil/water interface of the S1570 (0–0.500 wt%)-SC (0.50 wt%) system; variation of the slope value of the double logarithmic plot of dilatational complex modulus versus frequency with S1570 concentration (**b**). Different lowercase letters (a–f) above data points in subfigure (**b**) indicate significant differences (*p* < 0.05).

Figure 2a illustrated the impact of oscillation frequency (ranging from 0.02 to 0.20 Hz) on the oil/water interfacial composite modulus (*E*) of the S1570-SC system across various concentrations of S1570, using an amplitude of 10%.

It is observed that *E* increases with rising frequency (Figure 2a). At lower oscillation frequencies, there was sufficient time for surface-active substances in both the bulk phase and at the interface to exchange in response to changes in interfacial area, resulting in a lower *E*. Conversely, at higher frequencies, the oscillation period is shortened, reducing the time available for emulsifier molecules to undergo structural reorganization at the interface. The rapid change in the specific area of the interface enhances intermolecular interactions, leading to a higher E being exhibited [27,28].

The *E* exhibited an initial increase followed by a decrease as the concentration of S1570 varied. At a S1570 concentration of 0.010 wt%, *E* reached its maximum value, with a notable decline observed upon further increases in S1570 concentration. For instance, at an oscillation frequency of 0.20 Hz and a S1570 concentration of 0.001 wt%, the *E* of the stabilized interfacial layer in the S1570-SC system was 15.9 mN/m. Subsequently, as the S1570 concentration rose to 0.010 wt%, the *E* of the system increased to 32.8 mN/m. However, with a further increase in S1570 concentration to 0.500 wt%, the *E*-value of the S1570-SC system decreased to 15.3 mN/m.

In the absence of S1570, sodium caseinate, functioning as a linear macromolecular protein, inadequately covered the interface, resulting in weak intermolecular interactions and consequently a low *E*-value [29]. Upon the addition of S1570, acting as a small molecule nonionic emulsifier, it filled the void between the macromolecular proteins, thereby increasing the concentration of interfacial active substances. This augmentation enhanced intermolecular interactions at the interface during oscillation, resulting in a higher *E*-value. However, as the concentration of S1570 increased further, competitive adsorption between S1570 and SC took place at the interface [30]. Eventually, the dominance shifts to the small molecule S1570, leading to weaker interfacial intermolecular interactions. Consequently, the small molecule emulsifier can swiftly exchange between the bulk phase and the interfacial phase, reducing the interfacial tension gradient and resulting in a decrease in *E* [31,32].

Figure 2b illustrated the slope values of the double logarithmic linear fitting curve depicting the oil/water interfacial expansion complex modulus (*E*, mN/m) against frequency (Hz) for the S1570-SC system. The magnitude of the slope values reflects the viscoelastic nature of the interface. A slope value greater than 0.5 suggests that the expansion complex modulus primarily depends on the diffusion rate of the surface-active substance between the interface and the bulk phase, characteristic of reversible adsorption behavior of small molecule emulsifiers at the interface. Conversely, when the slope value approaches 0, the interface exhibits a completely elastic response, with the expansion complex modulus determined by the interaction between the surface-active substances adsorbed at the interface.

As the concentration of S1570 increased from 0 to 0.005 wt%, the slope decreased from 0.29 to 0.10, indicating that the interface displayed more elastic properties at this stage. This was attributed to the co-adsorption of low concentrations of S1570 and SC at the interface, enhancing molecular interactions and improving the elastic properties of the interface. Subsequently, as the concentration of S1570 rose to 0.500 wt%, the slope increased to 0.55, surpassing 0.5. At this point, the interfacial complex modulus was primarily influenced by molecular exchange between the interfacial phase and the bulk phase, typical of the interfacial behavior of small molecule emulsifiers. This suggests that S1570 dominated the interface at this concentration.

Figure 2c depicted the change in the loss angle with frequency for the S1570-SC interface. When the concentration of S1570 is below 0.010 wt%, an increase in S1570 concentration led to a significant decrease in the loss angle, indicating that the interface displayed more elastic properties. However, when the concentration of S1570 exceeds 0.010 wt%, further increases in concentration resulted in an apparent rise in the loss angle. At this stage, the S1570-SC interface system exhibited more viscous properties.

##### Amplitude Sweeping

**Figure 3:**
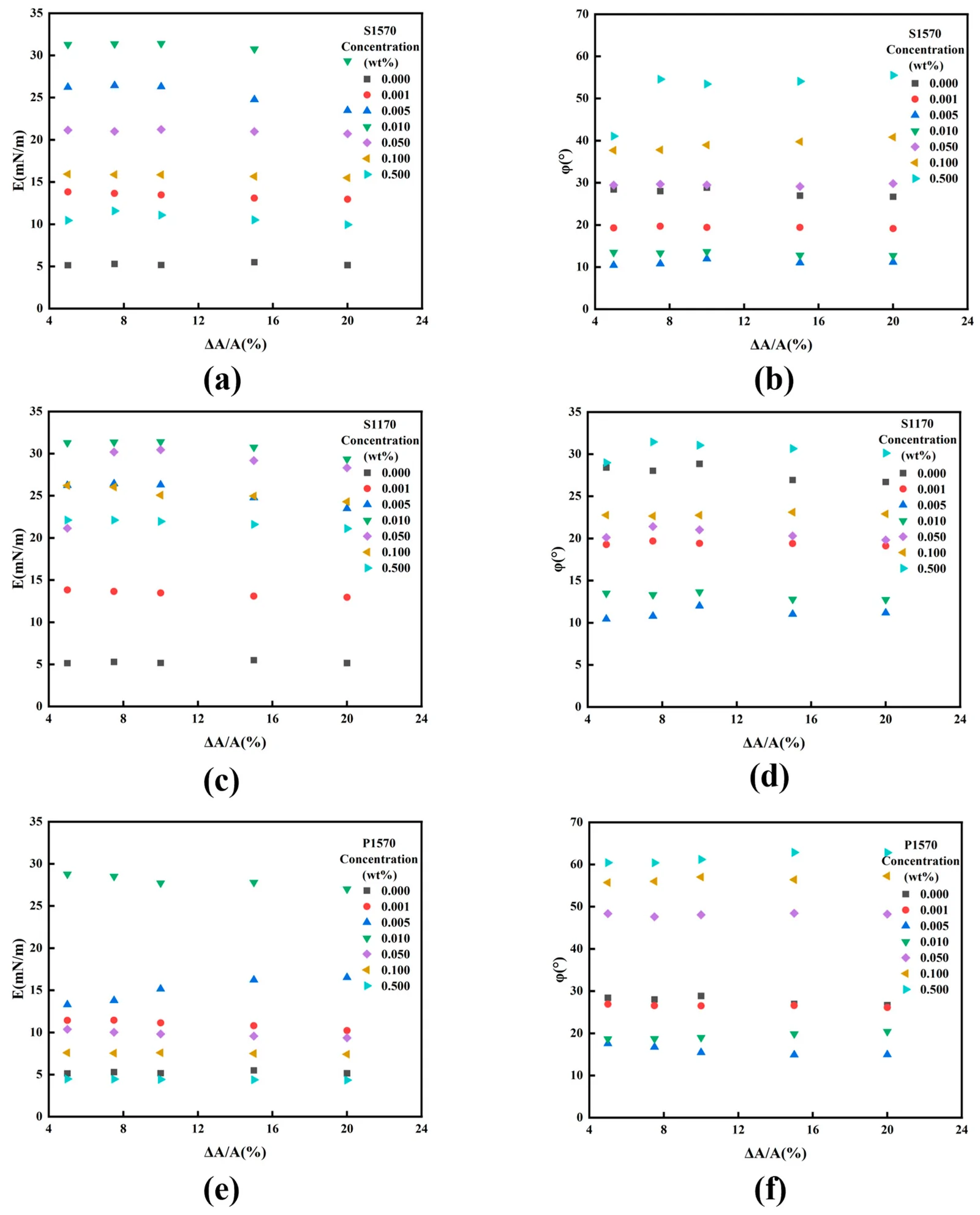
Complex interfacial dilatational modulus (**a**,**c**,**e**) and loss angle (**b**,**d**,**f**) as a function of amplitude for S1570–SC and S1170–SC systems. (**a**,**b**) S1570–SC; (**c**,**d**) S1170–SC; (**e**,**f**) P1570–SC. SE concentration: 0–0.500 wt%; SC: 0.50 wt%.

Figure 3a,b illustrate the impact of amplitude (ranging from 5% to 20%) on the interfacial composite modulus (*E*) and loss angle at various S1570 concentrations, with an oscillation frequency of 0.1. Interestingly, no significant effect of amplitude on either the interfacial composite modulus or loss angle is observed. This suggests that within the amplitudes examined in this study, changes in amplitude do not significantly alter the microstructure of the interfacial layer [33,34].

Consistent with the findings from frequency scanning, when the S1570 concentration is below 0.010 wt%, increasing the S1570 concentration tends to elevate the interfacial composite modulus while decreasing the loss angle of the system. Conversely, when the S1570 concentration exceeds 0.010 wt%, further increases in S1570 concentration lead to a reduction in the interfacial composite modulus and an increase in the loss angle of the system.

##### Lissajous Plots

The complex modulus of interfacial dilatation derived from frequency and amplitude scans is obtained through Fourier transformation based on the variation in interfacial tension. However, this approach neglects the original nonlinear information during the oscillation process. As a result, amplitude and frequency scans may not accurately capture the rheological response during oscillation [34,35]. To address this limitation and unveil the rheological characteristics of the interfacial dilatation and compression processes, further investigation is conducted using Lissajous plots between the change in interfacial tension and strain. These plots provide a more detailed understanding of the rheological properties of the system.

**Figure 4:**
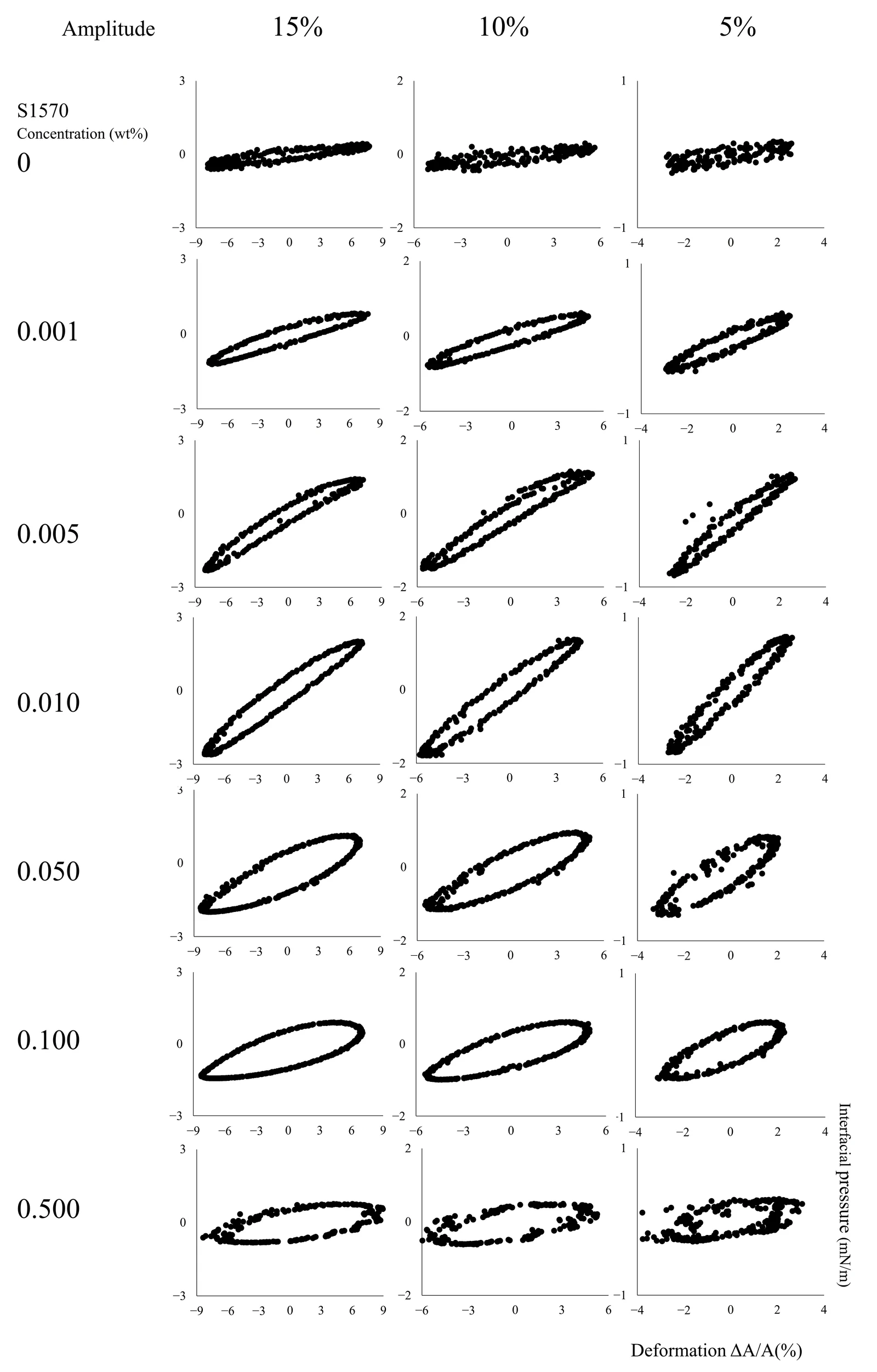
Lissajous plots obtained during amplitude sweeping (ΔA/A = 5%, 10% and 15%) of the oil/water interface stabilized by S1570 (0–0.500 wt%) and SC (0.50 wt%) mixtures.

Figure 4 displays the Lissajous graphs of sodium caseinate (SC) with varying concentrations of S1570 added at different amplitudes. In the absence of S1570, the Lissajous plots of SC at the oil/water interface exhibit relative linearity, with minimal changes in the slopes of the curves during expansion and compression. Upon increasing the concentration of S1570 to 0.005 wt%, the curves demonstrate an increase in the slope of interfacial pressure decrease during compression (lower part of the curve, from right to left), indicating the presence of strain hardening. Conversely, there is a decrease in the slope of interfacial pressure increase during dilatation (upper part of the curve, from left to right), indicating strain softening at the interface. At low concentrations, S1570 forms a more rigid interfacial network structure with sodium caseinate by filling the voids between protein molecules [29]. During compression, the interaction between the protein and the small molecule emulsifier intensifies, leading to strain hardening behavior. Conversely, during expansion, the rigid network structure formed by the protein and the small molecule emulsifier is disrupted, resulting in strain softening [3].

When the concentration of S1570 exceeds 0.010 wt%, the slope of the curve decreases in the decrease in interfacial pressure during compression, indicating the presence of strain softening at the interface. With increasing S1570 concentration, competitive adsorption of S1570 and SC occurs at the interface, gradually leading to the dominance of small molecule emulsifiers. Consequently, during compression, small molecule emulsifiers or proteins are easily desorbed from the interface, disrupting the initially formed interfacial layer and resulting in compression softening.

In summary, the hypothetical mechanism diagram illustrated in Figure S1 presents a proposed interpretation of the concentration-dependent interfacial behavior. In the absence of S1570 (Figure S1A), the network structure formed by SC at the interface may be relatively loose, resulting in a low interfacial dilatation complex modulus (*E*). During oscillation, compression and expansion have minimal influence on its interfacial network structure, displaying relative linearity. Upon increasing the S1570 concentration to 0.005 wt% (Figure S1B), S1570 and SC coexist at the interface, which is consistent with the formation of a denser interfacial network structure exhibiting more elastic properties and a higher *E*. Compression may enhance intermolecular interactions, resulting in strain-hardening behavior. As the S1570 concentration further increases to 0.050 wt% (Figure S1C), competitive adsorption between S1570 and SC occurs at the interface. Gradually, the small molecule emulsifier S1570 dominates the interface, which may disrupt the original dense structure and reduce the *E*-value. During oscillation, the interfacial structure is vulnerable to damage, exhibiting strain softening. With a further increase in S1570 concentration to 0.500 wt% (Figure S1D), the small molecule emulsifier completely dominates the interface. The interface displays typical properties of small molecule emulsifiers, characterized by increased viscosity and a lower *E*-value. During oscillation, viscous properties prevail, and the small molecule emulsifier can rapidly exchange between the interface and the bulk phase, facilitating the formation of new interfacial layers. These proposed interfacial structures represent plausible interpretations based on the interfacial tension and rheological results and were not directly observed at the molecular level.

#### 3.1.3. Effect of Sucrose Ester Types on Interfacial Rheological Properties

In the preceding section, the impact of sucrose ester S1570 concentrations on interfacial properties was investigated through frequency sweeping, amplitude sweeping, and Lissajous-plot analysis. In this section, a similar methodology was employed to analyze the influence of different sucrose ester species on the rheological properties of the oil/water interface. Specifically, the oil/water interfacial properties of the S1170-SC and P1570-SC systems were examined, respectively.

##### Frequency Scanning

Figure S2A,B depict the variation curves of the stabilized interfacial expansion complex modulus (*E*) with oscillation frequency and concentration for the S1170-SC and P1570-SC systems, respectively, at an amplitude of 10%. Similarly to the findings from the S1570 experiments, an increase in oscillation frequency led to an increase in *E*. As the sucrose ester concentration increased, the *E* of the stabilized oil/water interface for all three SE-SC composite systems initially increased and then decreased. The maximum *E* value was observed at a concentration of 0.010 wt% for all three sucrose esters. Beyond this concentration, *E* began to decline, with the S1170-SC system showing a slower decrease in *E*, while the P1570-SC system exhibited the most rapid decline. For instance, at a sucrose ester concentration of 0.100 wt% and an oscillation frequency of 0.2 Hz, the *E* of the S1570-SC system was 20.6 mN/m, the *E* of the S1170-SC system remained higher at 30.3 mN/m, while the *E* of the P1570-SC system dropped to just 12.0 mN/m.

The varying effects of different types of sucrose esters on the interfacial expansion modulus of the SE-SC system may be attributed to their hydrophobicity. The hydrophobicity of sucrose esters tends to increase with the elongation of fatty acid chains and higher degrees of esterification [4]. The S1170 contains more polyesters, which have higher hydrophobicity, leading to stronger intermolecular hydrophobic interactions during competitive adsorption with proteins at the interface, thereby maintaining a higher *E* [18]. In contrast, sucrose ester P1570 contains more short-chain sucrose palmitate, resulting in weaker intermolecular hydrophobic interactions during competitive adsorption with proteins, which contributes to the observed decrease in *E*.

Figure S2C,D present the slope values of the double-logarithmic linear fitting curves for the stabilized oil/water interfacial dilatational composite modulus (*E*, mN/m) versus frequency (Hz) for the S1170-SC and P1570-SC systems, respectively. The slopes for all three SE-SC systems initially increased and then decreased. At sucrose ester concentrations above 0.050 wt%, the slope of the S1170-SC system was lower, while the slope of the P1570-SC system was higher. Even at a concentration of 0.500 wt%, the slope for the S1170-SC system was 0.45, remaining below 0.50, indicating that the interface retained elastic properties. This may be due to stronger intermolecular interactions at the interface in the S1170-SC system, making it difficult for rapid exchange between the interface and the bulk phase during oscillation, thus exhibiting more elastic characteristics. But, the slope for the P1570-SC system reached as high as 0.57 at a concentration of 0.050 wt%, exceeding 0.50. In the P1570-SC system, the weaker intermolecular interactions at the interface allowed for rapid exchange between the bulk phase and the interface during oscillation, indicating more viscous properties.

Figure S2E,F show the variation of the loss angle with oscillation frequency for the S1170-SC and P1570-SC systems at different sucrose ester concentrations. Similarly to the S1570-SC system, the loss angle decreased and then increased with rising sucrose ester concentration. However, unlike the S1570-SC system, the loss angle for the S1170-SC system remained below 45° at all sucrose ester concentrations (0–0.500 wt%), indicating more elastic properties. In contrast, the loss angle for the P1570-SC system exceeded 45° at a concentration of 0.050 wt%, indicating a transition to more viscous properties.

##### Amplitude Scanning

Figure 3c–f illustrates the effect of oscillation frequency (0.1 Hz), sucrose ester concentration, and amplitude on E and loss angle of the S1170-SC and P1570-SC systems. Consistent with the results observed in the amplitude scans for S1570, the interfacial composite modulus and loss angle did not show significant variation across different amplitudes. This suggests that within the amplitude range studied in this paper (5–20%), changes in amplitude do not significantly impact the structure of the interfacial layer.

##### Lissajous Plots

Figures S3 and S4 show the Lissajous graphs between interfacial tension change and strain for the S1170-SC and P1570-SC systems with the addition of different concentrations of sucrose ester, respectively.

When the sucrose ester concentration is 0.010 wt%, the slopes of the Lissajous plots of the P1570-SC (Figure S4) and S1570-SC systems (Figure 4) already show a significant decrease during compression, exhibiting strain softening, while the slope of the Lissajous curve of the S1170-SC system (Figure S3) still increases, exhibiting strain hardening. This is due to the fact that, in the S1170-SC system, the intermolecular interactions at the interface are stronger. During compression, it is difficult for molecules to exchange in the bulk and interfacial phases, exhibiting stronger rigidity.

When the sucrose ester concentration was higher than 0.100 wt%, the Lissajous curve of the P1570-SC stabilized interface was narrower and more symmetric, and when oscillated, there was no obvious softening or hardening behavior. It may be due to the fact that P1570 can undergo rapid exchange at the interface and the bulk phase to compensate for the perturbation of interfacial tension due to the change in specific surface area.

In summary, the oil/water interfacial properties of the SE-SC system were affected by the esterification degree of sucrose esters and the length of fatty acid chains. S1170 contained more long-chain polyesters, which had stronger intermolecular hydrophobic interactions, and still showed higher viscoelasticity when it and protein adsorbed competitively at the interface; P1570 contained more short-chain monoesters, which had weaker intermolecular hydrophobic interactions and decreased rapidly when it adsorbed competitively with protein.

### 3.2. Emulsion Properties

**Figure 5:**
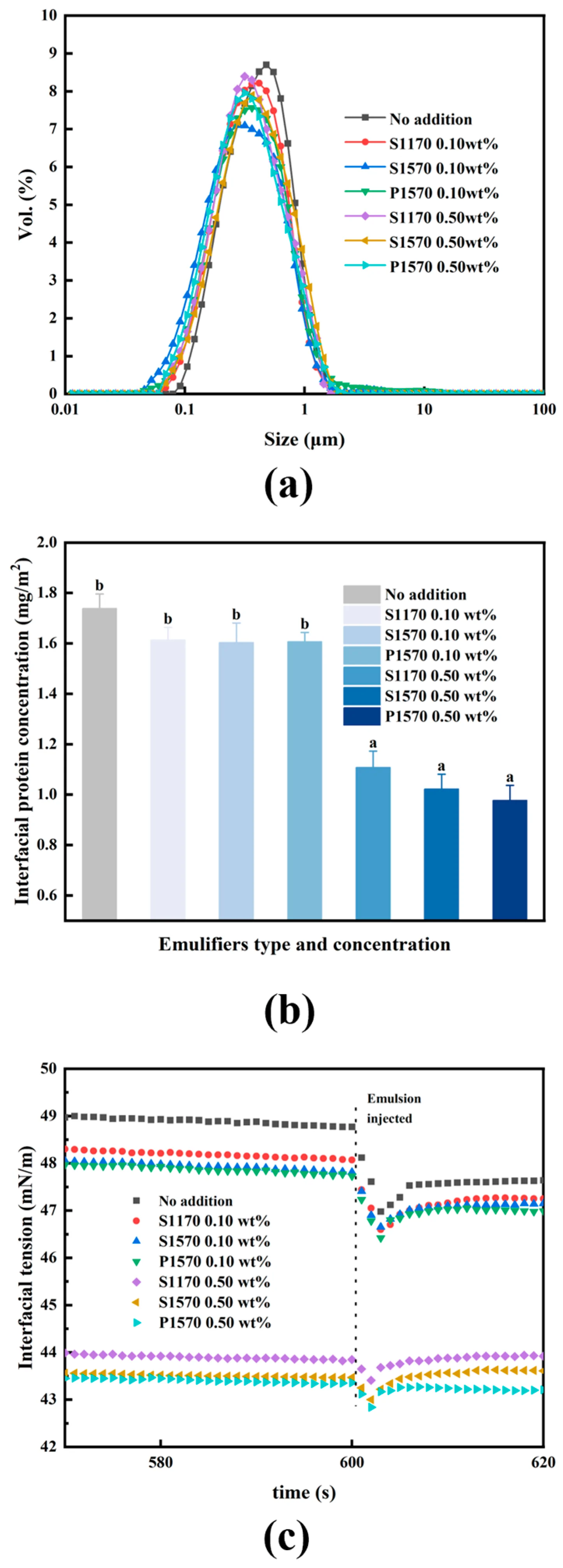
Effect of sucrose esters concentrations and types on droplets size distribution (**a**) and interfacial protein concentration (**b**) of emulsions. Air/water interfacial tension changes for serum phase after emulsion injection (**c**). Different letters a and b are statistically significantly different (*p* < 0.05).

Referring to previous studies, and aiming to minimize the impact of particle size on the quality of whipped cream [36,37], this section focuses on understanding how changes in interfacial properties affect the quality of whipped cream. To achieve this, emulsions with similar particle sizes and single-peak distributions were prepared by varying homogenization pressure (Figure 5a).

#### 3.2.1. Interface Protein Concentration

Figure 5b shows the interfacial protein concentrations of emulsions containing different concentrations and types of hydrophilic sucrose esters. Without the addition of sucrose ester, the interfacial protein concentration of the emulsions was 1.74 mg/m^2^. When 0.10 wt% sucrose ester was added, the interfacial protein concentration remained largely unchanged. However, when the sucrose ester concentration was increased to 0.50 wt%, the interfacial protein concentrations of the emulsions prepared with S1170, S1570, and P1570 were reduced to 1.10 mg/m^2^, 1.02 mg/m^2^, and 0.99 mg/m^2^, respectively.

When 0.10 wt% sucrose ester was added, both sucrose ester and sodium caseinate coexisted at the interface, resulting in no significant change in interfacial protein content. Droplet shape analysis revealed that at this concentration, sucrose ester and sodium caseinate competitively adsorbed at the interface, with sucrose ester becoming dominant. However, in the actual emulsion system, 0.10 wt% sucrose ester did not significantly affect the adsorption of sodium caseinate at the interface, so the interfacial protein concentration remained stable. This discrepancy arises because, in droplet method experiments, the sucrose ester adsorbs only on the surface of a droplet with a volume of about 5 μL, resulting in a relatively small specific surface area at the oil/water interface. In contrast, the actual emulsion has smaller fat globules with a larger surface area, where the sucrose ester adsorbs over a much larger oil/water boundary. As a result, the sucrose ester concentration at the interface in the actual system is much lower than in the simulated system, which explains why 0.10 wt%. Zhang et al. [32] observed a similar phenomenon in a study of the interaction between Tween 20 and bovine serum albumin in a foam system, where the concentration of Tween 20 that provided the best foam stability in droplet shape analysis was much lower than the concentration required for optimal stability in the actual foam system.

When the sucrose ester concentration reached 0.50 wt%, the higher concentration of sucrose ester competed more effectively with sodium caseinate at the interface. The sucrose ester, having higher interfacial activity, displaced the sodium caseinate, resulting in a lower concentration of protein at the interface. The interfacial protein concentrations of emulsions prepared with different sucrose esters showed no significant differences, suggesting that the ability of the three sucrose esters to displace interfacial proteins is quite similar. Studies on the competitive adsorption of Tween emulsifiers and proteins at the interface have found that the ability of hydrophilic small molecule emulsifiers to displace interfacial proteins is not significantly influenced by changes in their lipophilic groups [38,39].

#### 3.2.2. Adsorption of Fat Globules at the Air/Water Interface

After 600 s of equilibration, the air/water interfacial tension (γ_AW_) of the supernatant of the emulsion with 0.50 wt% sucrose ester was significantly lower than that of the supernatant of the emulsion with 0.10 wt% and no sucrose ester added (γ_AW_) (Figure 5c). For example, the γ_AW_ of the supernatant of the emulsion without sucrose ester was 48.77 mN/m; the γ_AW_ of the supernatant of the emulsion with 0.10 wt% S1170 decreased to 48.07 mN/m; and the γ_AW_ of the supernatant of the emulsion with 0.50 wt% S1170 decreased to 43.85 mN/m. The reason for the decrease in γ_AW_ of the supernatant of 0.50 wt% S1170 was the increase in the concentration of the protein in the liquid phase.

After injection of the emulsion, a significant decrease in γ_AW_ at re-equilibration was observed in the supernatant of all 0.10 wt% sucrose ester samples, whereas there was no significant change in γ_AW_ for the 0.50 wt% sucrose ester samples. The addition of 0.50 wt% sucrose esters increased the liquid-phase protein content of the supernatant, and after 600 s of equilibration, a higher concentration of protein was adsorbed on the air/water interface, which could prevent the fat globules from entering the air/water interface through spatial site resistance and electrostatic repulsion, so that the γ_AW_ did not change significantly after emulsion injection. In contrast, the content of protein adsorbed at the air/water interface in the supernatant with 0.10 wt% sucrose ester added was less, and the fat globules could enter the air/water interface after emulsion injection, resulting in a decrease in γ_AW_.

### 3.3. Whipped Cream Quality

#### 3.3.1. Partial Coalescence Degree

**Figure 6:**
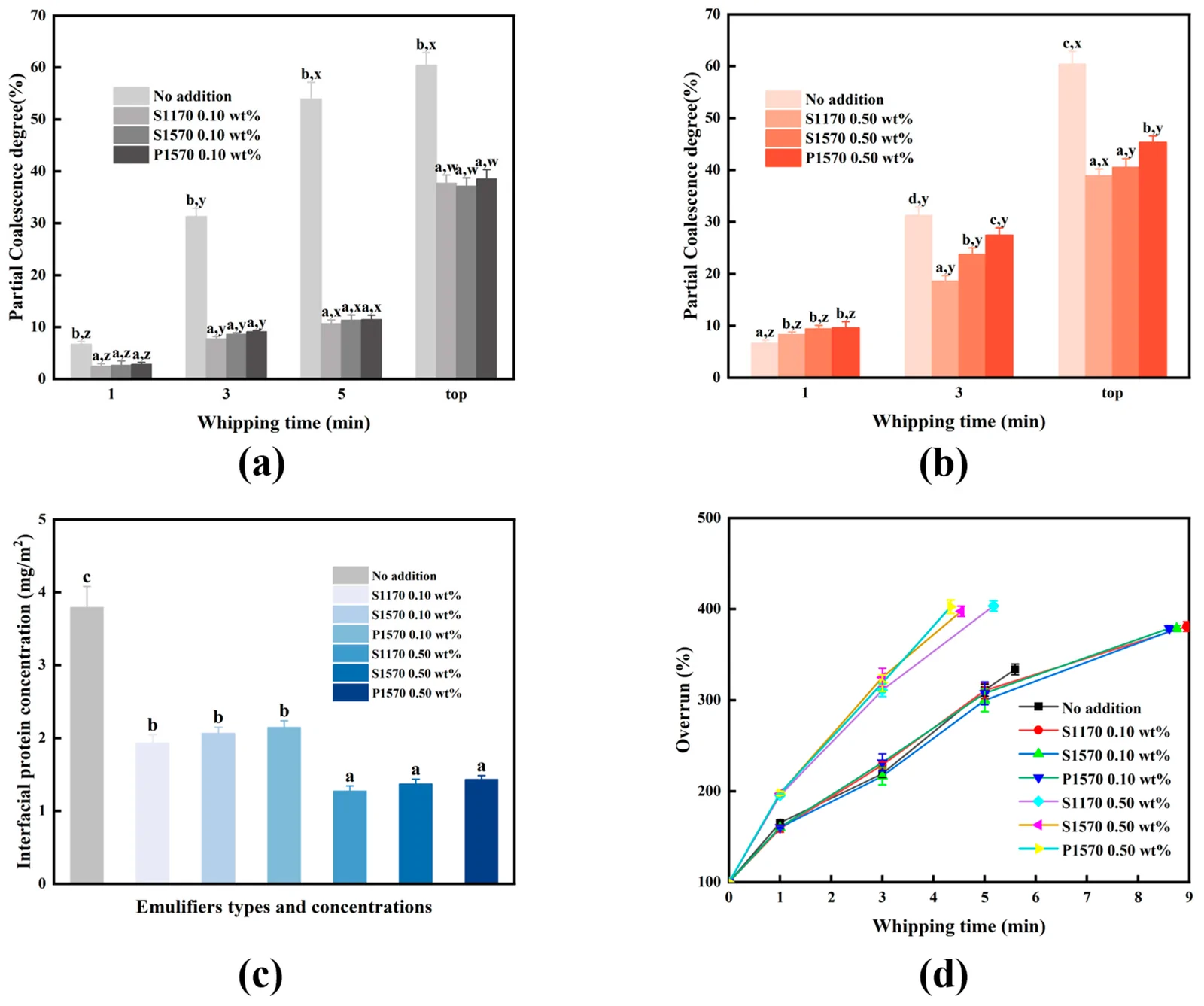
Effects of sucrose ester type and concentration on the degree of fat partial coalescence during whipping at (**a**) 0.10 wt% and (**b**) 0.50 wt%, (**c**) the interfacial protein concentration of whipped cream, and (**d**) the overrun during whipping. Different lowercase letters a–d indicate statistically significant (*p* < 0.05) differences between samples with different types of sucrose esters at the same churning time; different lowercase letters w–z indicate statistically significant (*p* < 0.05) differences between samples with different churning times at the same sucrose ester concentration.

Figure 6a shows the change in the rate of fat partial coalescence degree during whipping at a sucrose ester concentration of 0.10 wt%. The addition of 0.10 wt% sucrose ester significantly reduced the rate of partial coalescence during whipping. At the optimal whipping time, even though the whipping time of the whipped cream with 0.10 wt% sucrose ester was significantly longer than others, its partial coalescence degree was still lower. At a sucrose ester concentration of 0.10 wt%, the type of sucrose ester did not have a significant effect on the fat partial agglomeration rate.

Without the addition of sucrose esters, the protein-only interface was less viscoelastic and was easily punctured by fat crystals during whipping, resulting in a faster rate of fat globules coalescence. When 0.10 wt% sucrose ester was added, the sucrose ester and sodium caseinate together formed a more viscoelastic interfacial layer on the surface of the fat globules, which was more difficult to be punctured by fat crystals inside the fat globules, leading to a lower rate of fat partial coalescence degree.

Figure 6b showed the fat partial coalescence degree during whipping when the sucrose ester concentration was 0.50 wt%. At the 1st min of whipping, the fat partial coalescence degree of the whipped cream with 0.50 wt% sucrose esters was significantly higher than that of the unadded sample; however, at the 3rd min of whipping and the optimal whipping time, the fat partial coalescence degree of the sucrose ester-added whipped cream was lower than that of the samples with no sucrose ester added. Among the three whipped creams with different sucrose esters, the highest degree of partial coalescence was observed in the P1570-added cream samples and the lowest in the S1170 samples.

At the initial stage of whipping (1 min), shear-induced partial coalescence predominantly occurred. The competitive adsorption of 0.50 wt% sucrose ester and sodium caseinate on the surface of the fat globules compromised the integrity of the interfacial layer, reducing its viscoelasticity. This weakening made it easier for fat crystals within the globules to break through the interfacial layer during whipping, thereby accelerating the rate of fat partial coalescence. As the interfacial protein concentration decreased, the spatial resistance and electrostatic repulsion on the surface of the fat globules also diminished, further speeding up the shear-induced partial coalescence of the fat during the whipping process [40].

As whipping progressed to 3 min, the surface area of the bubbles increased, leading to surface-mediated partial coalescence becoming the dominant process. Fat globules from samples with higher sucrose ester concentrations found it more difficult to reach the air/water interface, resulting in slower surface-mediated partial coalescence. This caused a lower rate of fat coalescence compared to samples without added sucrose ester.

Interfacial rheological properties revealed that, under competitive adsorption, the interfacial complex modulus of oil/water stabilized by P1570-SC was the lowest, making it the most susceptible to disruption during whipping. Consequently, the whipping cream prepared with P1570-SC exhibited a higher coalescence rate during whipping. Conversely, the interfacial complex modulus of S1170-SC was higher, making it more resistant to internal crystallization, and the fat phase in creams prepared with S1170-SC showed a lower coalescence rate.

#### 3.3.2. Interfacial Protein Concentration

The interfacial protein concentrations of whipped creams with different sucrose ester concentrations are presented in Figure 6c. Interfacial protein concentration is jointly determined by the specific surface area (SSA) and the amount of protein adsorbed at the oil/water interface, and it exerts a vital influence on the stability and hardness of whipped cream. The interfacial protein concentration of whipped cream decreased markedly with the rising sucrose ester concentration. For instance, the interfacial protein concentration of whipped cream without sucrose ester was 3.79 mg/m^2^; this value dropped to 1.93 mg/m^2^ for the sample supplemented with 0.10 wt% S1170, and further decreased to 1.27 mg/m^2^ when 0.50 wt% S1170 was incorporated. At identical concentrations, no obvious differences in interfacial protein content were observed among cream samples formulated with different types of sucrose esters.

For whipped creams containing 0.10 wt% sucrose ester, the reduction in interfacial protein concentration can be mainly attributed to the low degree of fat partial coalescence, which yields a relatively large specific surface area of fat and consequently reduces the protein concentration per unit area in the final whipped cream. A further decline in interfacial protein concentration occurred in cream samples with 0.50 wt% sucrose ester, owing to competitive adsorption between high-concentration sucrose esters and proteins at the oil-water interface.

#### 3.3.3. Optimal Whipping Time

Table 2 shows the effect of different types and concentrations of sucrose esters on the optimal whipping time (t_op_) for whipped cream. The addition of 0.10 wt% sucrose ester significantly (*p* < 0.05) extended the t_op_ for whipped cream, whereas the addition of 0.50 wt% sucrose ester shortened it. Notably, the cream prepared with P1570 had the shortest t_op_, at 4.33 min.

The addition of 0.10 wt% sucrose ester slowed down the rate of fat coalescence during the whole whipping process, requiring more time to form the fat network structure necessary to stabilize the final whipped cream, thereby prolonging the t_op_. In contrast, when 0.50 wt% sucrose ester was added, the faster shear-induced partial coalescence accelerated the process, resulting in a shorter t_op_. Among the creams, the one prepared with P1570, which exhibited the fastest partial coalescence rate in the initial stage, had the shortest t_op_.

#### 3.3.4. Overrun

Figure 6d illustrates the overrun of whipping rate over time for different cream samples. The addition of 0.10 wt% sucrose ester did not significantly impact the initial whipping rate of the cream; however, as the t_op_ lengthened, the whipped cream prepared with this concentration exhibited a higher overrun by the end of the process (as seen in Table 2). Conversely, the addition of 0.50 wt% sucrose ester significantly increased the overrun per unit of time. Despite the shortened t_op_, the final whipping rate of the creams prepared with this concentration was significantly higher than that of other creams. The type of sucrose ester used did not have a significant effect on the whipping rate at the same concentration.

The overrun of whipped cream is influenced by factors such as the rate of fat partial coalescence and the liquid phase protein concentration [41]. Since there was no significant difference in the liquid phase protein concentration between the 0.10 wt% sucrose ester emulsions and those without added sucrose esters, the whipping rate remained similar at the same churning time. In samples containing 0.10 wt% sucrose ester, the fat partial coalescence occurs more slowly, leading to longer whipping and aeration times, which allowed for more air incorporation and a significantly higher overrun at the t_op_. In contrast, the 0.50 wt% sucrose ester cream samples had a higher liquid phase protein concentration, resulting in a significantly higher overrun for the same whipping time. Consequently, the final overruns were higher, even with a shorter t_op_.

#### 3.3.5. Firmness

As shown in Table 2, the firmness of whipped cream decreased significantly with increasing sucrose ester concentration, whereas no significant differences were observed among the three sucrose ester types at the same concentration. Whipped cream firmness is influenced by several factors, including interfacial protein concentration, overrun, and the degree of fat partial coalescence. In the absence of sucrose esters, the whipped cream exhibited a relatively high degree of partial coalescence and interfacial protein concentration, together with a lower overrun, which was consistent with the formation of a denser internal network and a higher firmness.

At 0.10 wt% sucrose ester, the reductions in partial coalescence and interfacial protein concentration, combined with the higher overrun, were associated with a less compact network and consequently lower firmness. At 0.50 wt%, although the degree of fat partial coalescence increased to some extent, the partially coalesced fat aggregates appeared less able to associate effectively with the air-bubble surfaces, while the interfacial protein concentration was the lowest. These changes were consistent with the weakest internal network and, therefore, the lowest firmness.

#### 3.3.6. Microstructure

**Figure 7:**
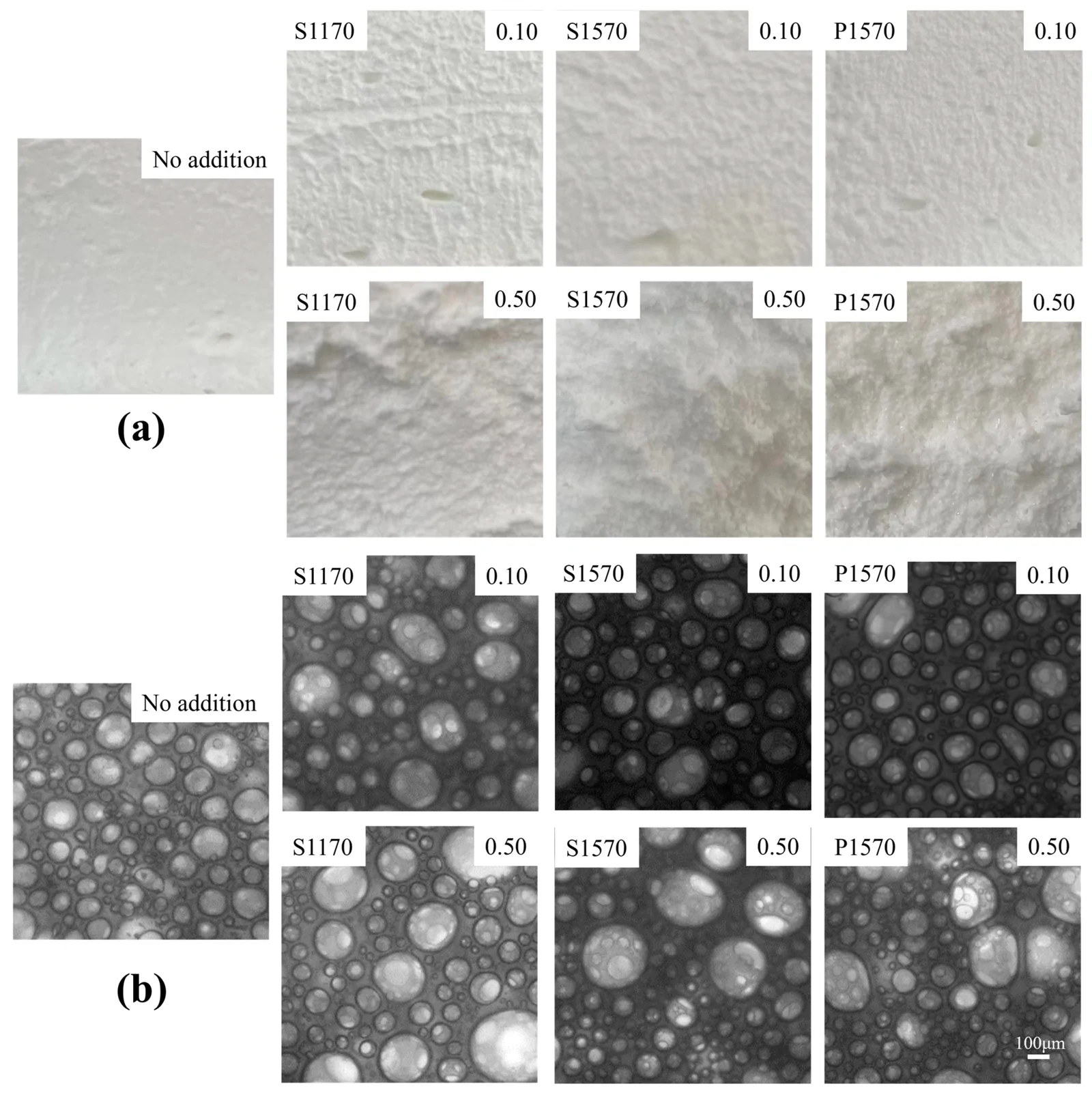
(**a**) Cut-surface appearance of whipped cream after storage at 25 °C for 12 h. SE, sucrose ester. (**b**) Optical micrographs of whipped cream prepared with different sucrose ester types and concentrations. The images show differences in bubble size, distribution, and structural uniformity among the treatments. Images were obtained at [×100 magnification], and the scale bar represents [100 μm].

The distribution of air bubbles in whipped cream was observed using optical microscopy, with microscopic images of whipped creams prepared with varying types and concentrations of sucrose esters displayed in Figure 7a. At the same concentration, no significant differences in the microstructure of whipped creams were observed among the different types of sucrose esters used. However, increasing the concentration of sucrose ester notably increased the number of large air bubbles in the cream. When the sucrose ester concentration was 0.10 wt%, the prolonged whipping time contributed to a higher number of large air bubbles within the cream system. In contrast, at a concentration of 0.50 wt%, the higher protein concentration in the liquid phase allowed for quicker adsorption onto the surface of air bubbles during the whipping process, leading to an even greater presence of large air bubbles.

#### 3.3.7. Stabilities

The stability of whipped cream can be assessed by examining the serum loss during storage (as shown in Table 2) and the appearance of the cut surface (Figure 7b). An increase in sucrose ester concentration led to a significant rise in water loss and a rougher cut surface structure. At a sucrose ester concentration of 0.10 wt%, the stability of whipped cream did not show much variation among the different sucrose esters. However, at a concentration of 0.50 wt%, there was a notable difference: the whipped cream made with sucrose ester S1170 had a relatively lower water yield of 22.2%, whereas whipped cream with P1570 had the highest water yield at 29.4% and the roughest cut surface structure.

Whipped cream stability was related to the network structure formed by the interfacial protein layer and the partial coalescence fat network. In the sample with 0.10 wt% sucrose ester, the partial coalescence degree is lower, and there are more internal large-sized bubbles, resulting in a looser network structure and reduced stability. In the sample with 0.50 wt% sucrose ester added, there were more internal large-size bubbles, and it was difficult for some of the fat globules to coalesce on the surface of the bubbles to stabilize the bubbles, which led to a significant decrease in their stability.

When the sucrose ester concentration was 0.50 wt%, the difference in cream stability might be related to the viscoelasticity of its interfacial layer. At high sucrose ester concentration, the interfacial layer stabilized by S1170-SC still had high viscoelasticity, resulting in higher stability of fat globule coalescence in whipped cream; the interfacial layer stabilized by P1570-SC had the lowest viscoelasticity, resulting in the worst stability of the final whipped cream.

Overall, S1170 provided the most balanced interfacial and whipping performance among the three sucrose esters, particularly at 0.50 wt%. Its relatively high interfacial viscoelasticity was associated with lower serum loss and better structural stability. S1570 generally exhibited intermediate behavior. In contrast, P1570 promoted faster early-stage fat partial coalescence and shortened the optimal whipping time, but its lower interfacial modulus and higher serum loss indicated poorer storage stability. Therefore, S1170 may be more suitable when a balance between whipping performance and storage stability is required, whereas P1570 may be advantageous for faster whipping but at the expense of final product stability.

## 4. Conclusions

This study demonstrated that the type and concentration of hydrophilic sucrose esters jointly regulated the interfacial properties and whipping performance of sodium-caseinate-stabilized cream systems. At 0.10 wt%, the rheological results were consistent with the formation of a mixed interfacial layer with enhanced viscoelasticity, accompanied by slower fat partial coalescence and increased overrun. At 0.50 wt%, stronger competitive adsorption reduced interfacial protein coverage, which was consistent with a weaker and more mobile interfacial film, faster early-stage partial coalescence, and poorer storage stability. Among the three sucrose esters, S1170 showed behavior consistent with stronger intermolecular interactions and provided the most balanced combination of interfacial viscoelasticity, whipping performance, and serum stability. S1570 exhibited intermediate behavior, whereas P1570 shortened the whipping time but resulted in lower interfacial modulus and greater serum loss. These interfacial differences were associated with the distinct whipping and stability characteristics of the cream systems. Together, these findings support a plausible structure–interface–whipping relationship and provide an interfacial basis for the rational selection of sucrose ester type and dosage in whipped cream formulations.

## Figures and Tables

**Table 1:** Formulations of the emulsions used for whipped cream preparation.

Treatment	Sucrose Ester Type	Sucrose Ester (wt%)	SC (wt%)	Stabilizers (wt%)	HPKO (wt%)	Corn Syrup (wt%)	Sucrose (wt%)	Glucose (wt%)	Water (wt%)
Control	—	0	0.50	0.40	14.0	6.2	5.0	18.0	Balance to 100
S1170-0.10	S1170	0.10	0.50	0.40	14.0	6.2	5.0	18.0	Balance to 100
S1170-0.50	S1170	0.50	0.50	0.40	14.0	6.2	5.0	18.0	Balance to 100
S1570-0.10	S1570	0.10	0.50	0.40	14.0	6.2	5.0	18.0	Balance to 100
S1570-0.50	S1570	0.50	0.50	0.40	14.0	6.2	5.0	18.0	Balance to 100
P1570-0.10	P1570	0.10	0.50	0.40	14.0	6.2	5.0	18.0	Balance to 100
P1570-0.50	P1570	0.50	0.50	0.40	14.0	6.2	5.0	18.0	Balance to 100

**Table 2:** The effect of sucrose ester types and concentrations on whipped cream properties.

SE Type and Concentrations (wt%)	*t_op_* (min)	Overrun (%)	Firmness (N)	Serum Loss (%)
No addition	5.59 ± 0.21 ^c^	333.7% ± 5.9% ^a^	2.29 ± 0.04 ^c^	10.3% ± 1.3% ^a^
S1170 0.10 wt%	8.95 ± 0.54 ^d^	381.0% ± 5.3% ^b^	1.95 ± 0.06 ^b^	15.6% ± 1.8% ^b^
S1570 0.10 wt%	8.76 ± 0.42 ^d^	378.6% ± 3.0% ^b^	1.96 ± 0.06 ^b^	18.0% ± 2.0% ^b^
P1570 0.10 wt%	8.61 ± 0.42 ^c^	378.9% ± 2.7% ^b^	1.98 ± 0.07 ^b^	17.8% ± 1.4% ^b^
S1170 0.50 wt%	5.17 ± 0.14 ^b^	403.4% ± 7.2% ^c^	1.73 ± 0.07 ^a^	22.2% ± 1.8% ^c^
S1570 0.50 wt%	4.54 ± 0.09 ^a^	397.5% ± 5.6% ^c^	1.69 ± 0.08 ^a^	26.5% ± 2.7% ^cd^
P1570 0.50 wt%	4.33 ± 0.24 ^a^	402.5% ± 7.4% ^c^	1.72 ± 0.05 ^a^	29.4% ± 1.8% ^d^

Different lowercase letters a–d in the same column indicate statistically significant (*p* < 0.05) differences between samples.

## Data Availability

The original contributions presented in the study are included in the article/Supplementary Materials, further inquiries can be directed to the corresponding authors.

## Supplementary Materials

The following supporting information can be downloaded at: https://www.mdpi.com/article/10.3390/foods15172995/s1, Figure S1. Proposed schematic interpretation of different concentrations (A—0, B—0.005 wt%, C—0.050 wt% and D—0.500 wt%) sucrose ester S1570 with SC at Oil/Water Interface; Figure S2. Variation of expansion complex modulus (A—S1170, B—P1570) and loss angle (E—S1170, F—P1570) with frequency for the stable oil/water interface of the SE (0–0.500 wt%)-SC (0.50 wt%) system; the slope values of the double logarithmic plots of the expansion complex modulus versus frequency vary with SE concentration (C—S1170, D—P1570); Figure S3. Complex interfacial dilatational modulus (A—S1170, B—P1570) and loss angle (C—S1170, D—P1570) as a function of amplitude for SE (0–0.500 wt%)-SC (0.50 wt%); Figure S4. Lissajous plots obtained during amplitude sweeps (ΔA/A = 5%, 10% and 15%) of the oil/water interface stabilized by S1170 (0–0.500 wt%) and SC (0.50 wt%) mixtures; Figure S5. Lissajous plots obtained during amplitude sweeps (ΔA/A = 5%, 10% and 15%) of the oil/water interface stabilized by P1570 (0–0.500 wt%) and SC (0.50 wt%) mixtures.

## Abbreviations

The following abbreviations are used in this manuscript:

ANOVA	Analysis of variance
SC	Sodium caseinate
SE	Sucrose ester
HLB	Hydrophilic-lipophilic balance
HPKO	Hydrogenated palm kernel oil
wt%	Weight percentage
rpm	Revolutions per minute
