# Supplementary material for: Whipped Cream Regulation by Hydrophilic Sucrose Esters: Interfacial Behavior and Whipping Properties
Source: Foods. 2026 Aug 26;15(17):2995. doi: 10.3390/foods15172995 (PMC13564632; doi:10.3390/foods15172995)
Supplement: Supplementary file 1 [file foods-15-02995-s001.zip › foods-4445959-supplementary.pdf]

Supplementary Materials:

Figure S1 Proposed schematic interpretation of different concentrations (A-0, B-0.005 wt%, C-0.050 wt% and D-0.500 wt%) sucrose ester S1570 with SC at Oil/Water Interface

Figure S2 Variation of expansion complex modulus (A-S1170, B-P1570) and loss angle (E-S1170, F-P1570) with frequency for the stable oil/water interface of the SE (0-0.500 wt%)-SC (0.50 wt%) system; the slope values of the double logarithmic plots of the expansion complex modulus versus frequency vary with SE concentration (C-S1170, D- P1570)

Figure S3 Complex interfacial dilatational modulus (A-S1170, B-P1570) and loss angle (C-S1170, D-P1570) as a function of amplitude for SE (0-0.500 wt%)-SC (0.50 wt%)

Figure S4 Lissajous plots obtained during amplitude sweeps ($\Delta A/A$ = 5%, 10% and 15%) of the oil/water interface stabilized by S1170 (0-0.500 wt%) and SC (0.50 wt%) mixtures.

Figure S5 Lissajous plots obtained during amplitude sweeps ($\Delta A/A$ = 5%, 10% and 15%) of the oil/water interface stabilized by P1570 (0-0.500 wt%) and SC (0.50 wt%) mixtures.

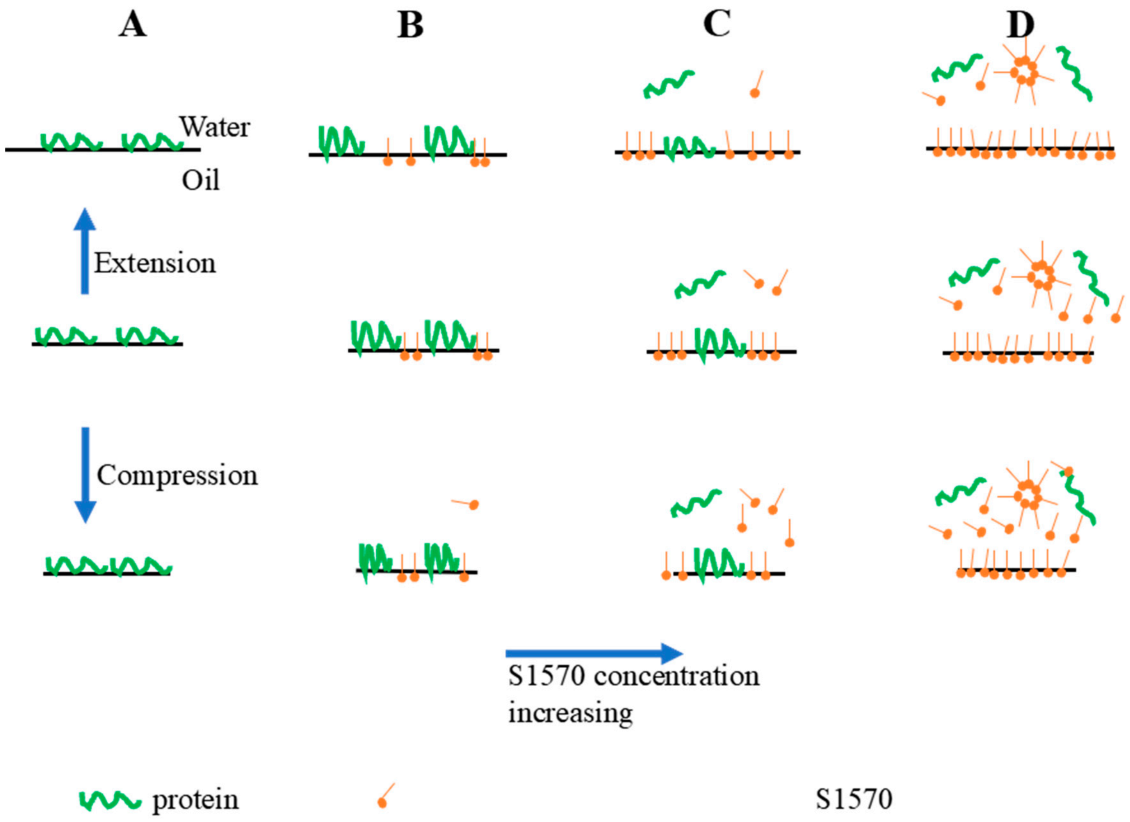

Figure S1. Proposed schematic interpretation of different concentrations (A-0, B-0.005 wt%, C-0.050 wt% and D-0.500 wt%) sucrose ester S1570 with SC at Oil/Water Interface

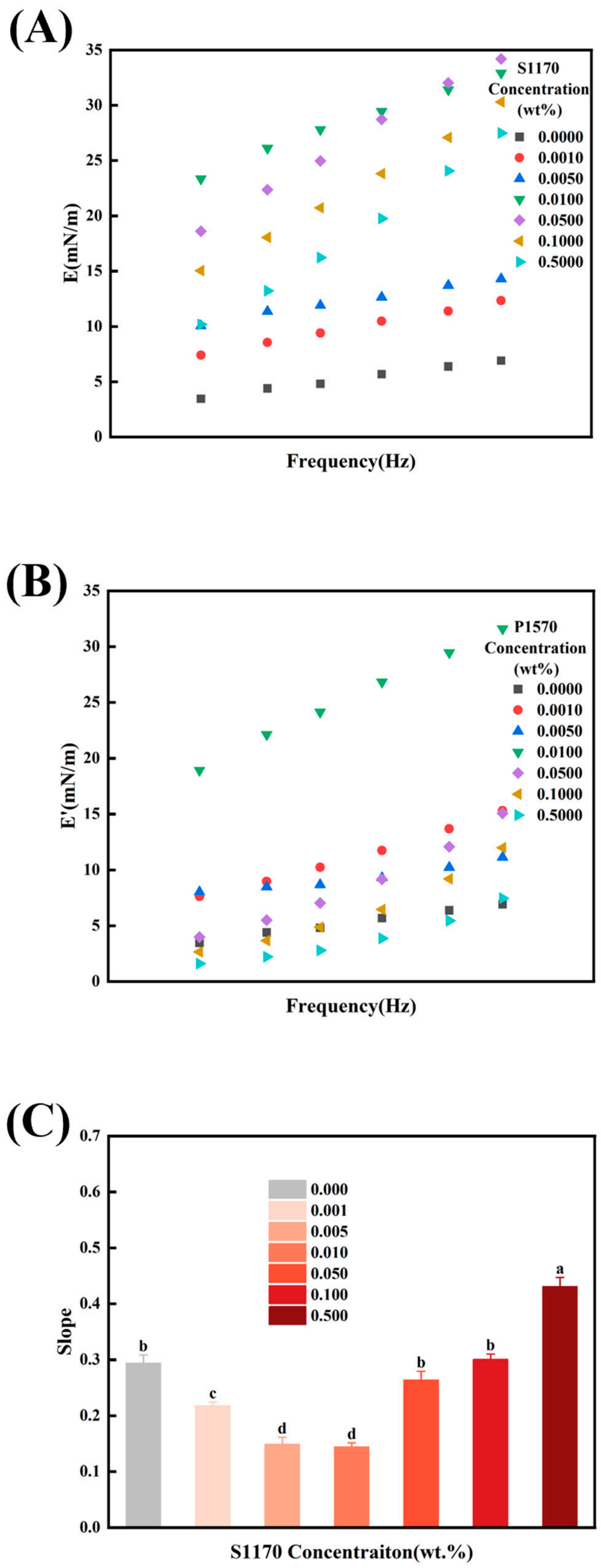

(A)
E(mN/m)
Frequency(Hz)
S1170
Concentration
(wt%)
0.0000
0.0010
0.0050
0.0100
0.0500
0.1000
0.5000
(B)
E'(mN/m)
Frequency(Hz)
P1570
Concentration
(wt%)
0.0000
0.0010
0.0050
0.0100
0.0500
0.1000
0.5000
(C)
Slope
S1170 Concentraiton(wt.%)
0.000
0.001
0.005
0.010
0.050
0.100
0.500
b
c
d
d
b
b
a

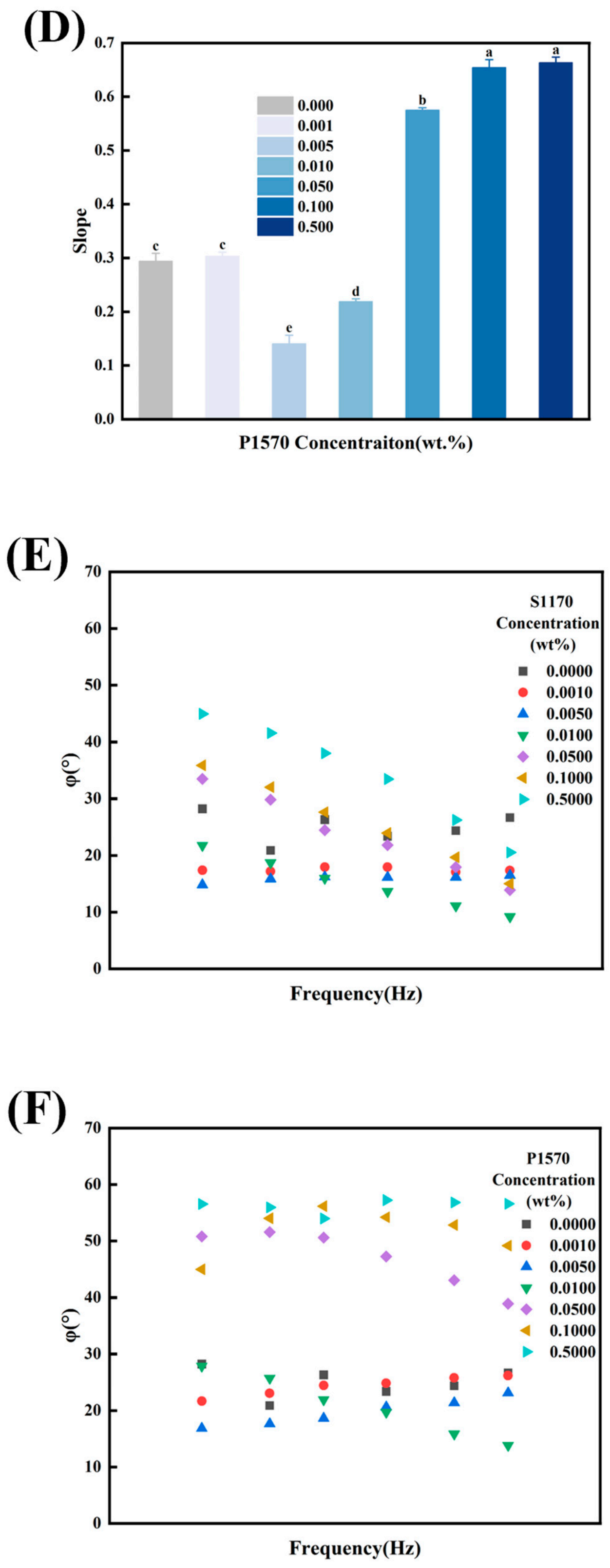

Figure S2 Variation of expansion complex modulus (A-S1170, B-P1570) and loss angle (E-S1170, F-P1570) with frequency for the stable oil/water interface of the SE (0-0.500 wt%)-SC (0.50 wt%) system; the slope values of the double logarithmic plots of the expansion complex modulus versus frequency vary with SE concentration (C-S1170, D- P1570)

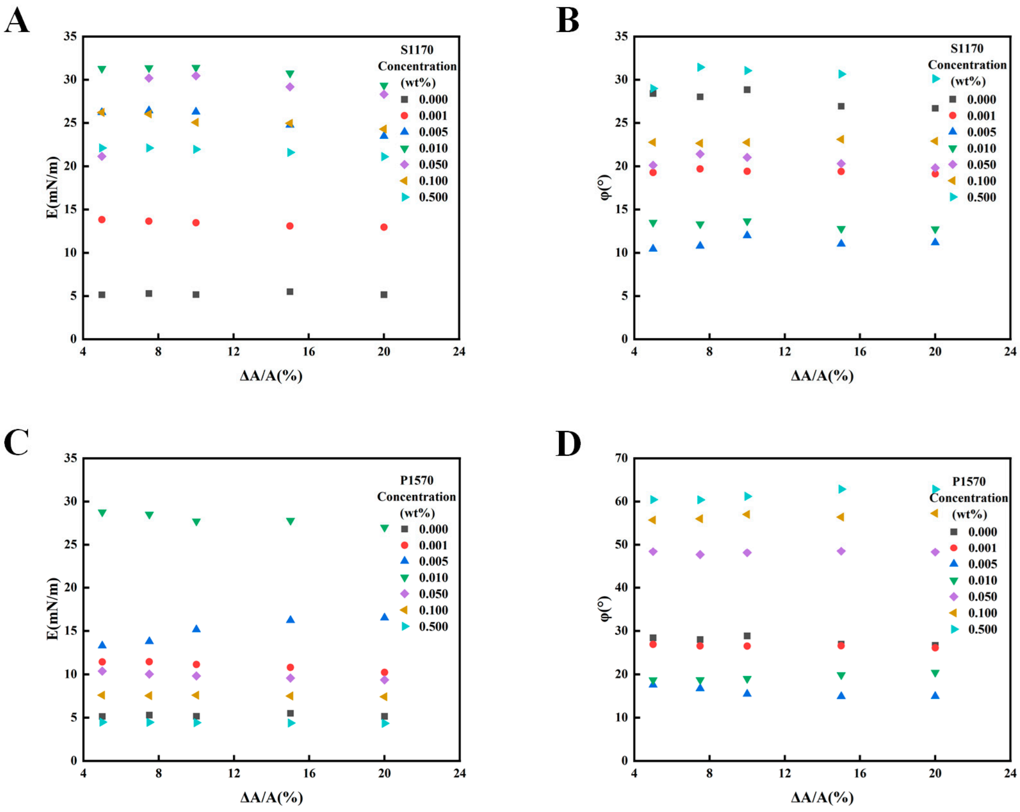

Figure S3 Complex interfacial dilatational modulus (A-S1170, B-P1570) and loss angle (C-S1170, D-P1570) as a function of amplitude for SE (0-0.500 wt%)-SC (0.50 wt%)

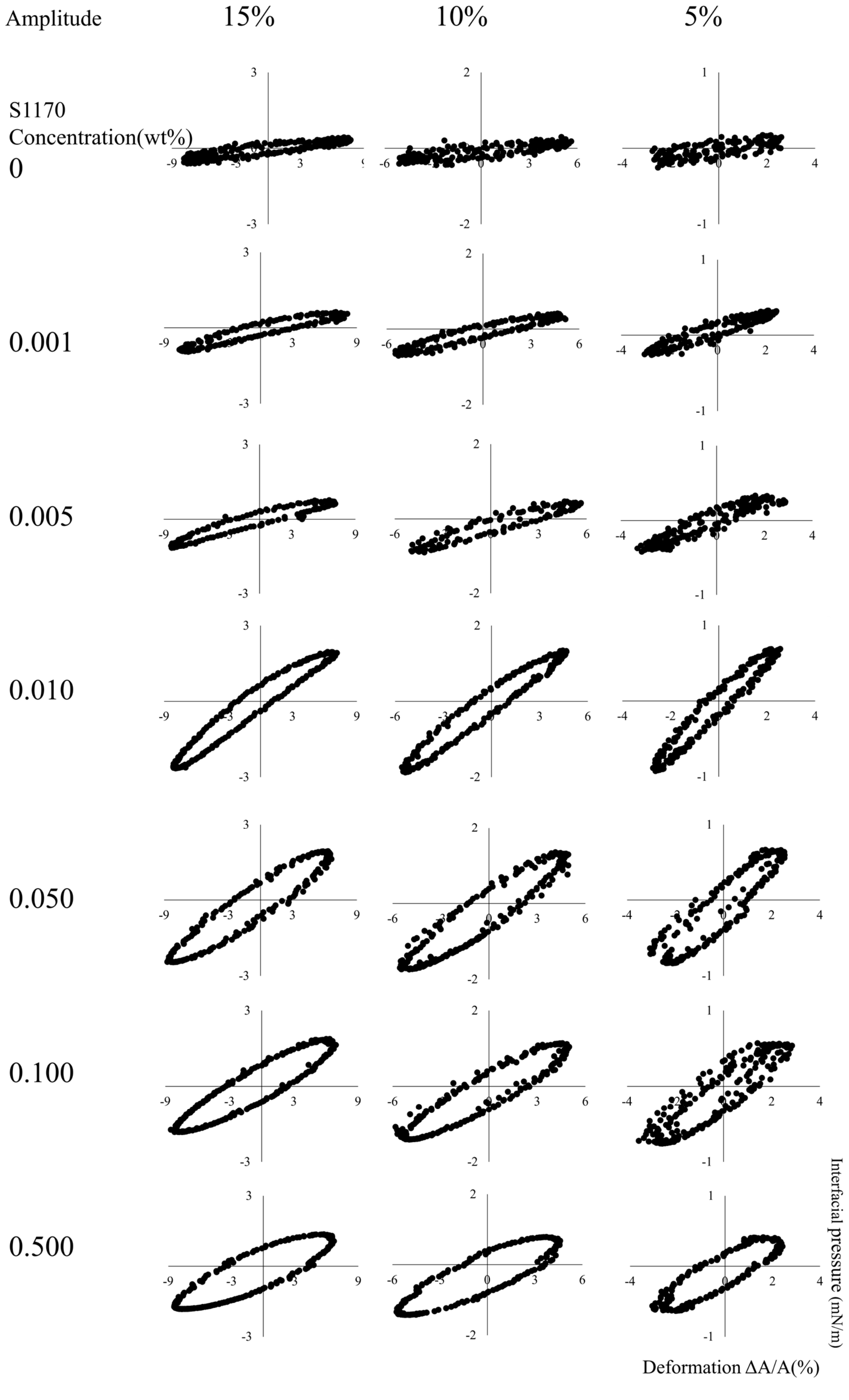

Figure S4 Lissajous plots obtained during amplitude sweeps (ΔA/A = 5%, 10% and 15%) of the oil/water interface stabilized by S1170 (0-0.500 wt%) and SC (0.50 wt%) mixtures.

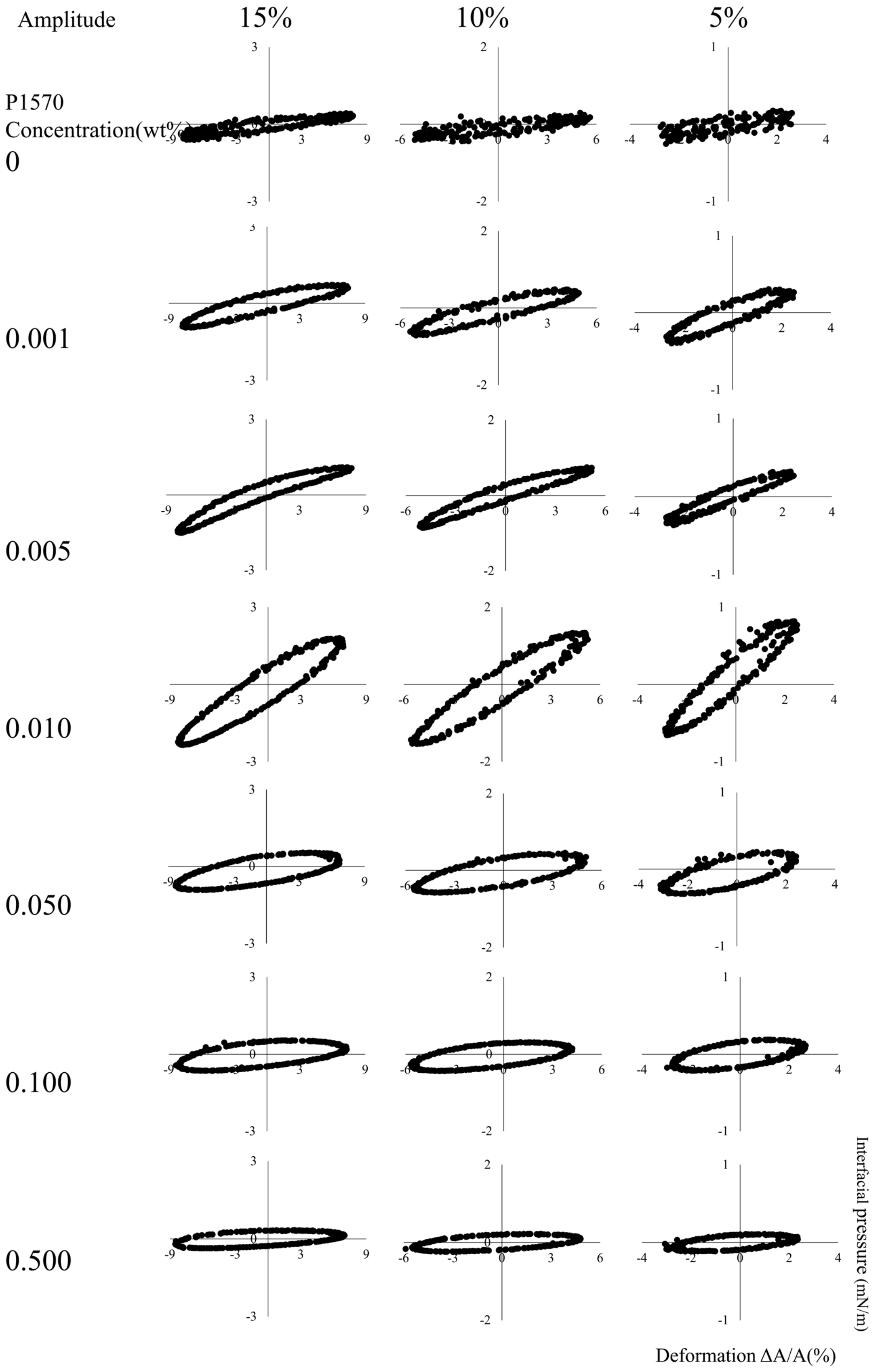

Figure S5 Lissajous plots obtained during amplitude sweeps (ΔA/A = 5%, 10% and 15%) of the oil/water interface stabilized by P1570 (0-0.500 wt%) and SC (0.50 wt%) mixtures.
